# Not Asian Anymore: Reconstruction of the History, Evolution, and Dispersal of the “Asian” Lineage of CPV-2c

**DOI:** 10.3390/v15091962

**Published:** 2023-09-20

**Authors:** Giovanni Franzo, Francesco Mira, Giorgia Schirò, Marta Canuti

**Affiliations:** 1 Department of Animal Medicine, Production and Health (MAPS), Padua University, 35020 Legnaro, Italy; 2 Istituto Zooprofilattico Sperimentale della Sicilia “A. Mirri”, 90129 Palermo, Italy; francesco.mira@izssicilia.it (F.M.); giorgia.schiro91@gmail.com (G.S.); 3 Department of Veterinary Science, University of Messina, Polo Universitario dell’Annunziata, 98168 Messina, Italy; 4 Department of Pathophysiology and Transplantation, Università degli Studi di Milano, 20122 Milan, Italy; 5 Coordinate Research Centre EpiSoMI (Epidemiology and Molecular Surveillance of Infections), Università degli Studi di Milano, 20122 Milan, Italy; 6 Centre for Multidisciplinary Research in Health Science (MACH), Università degli Studi di Milano, 20122 Milan, Italy

**Keywords:** canine parvovirus type 2, CPV-2, molecular epidemiology, *Protoparvovirus carnivoran1*, parvovirus, virus evolution

## Abstract

Variability has been one of the hallmarks of canine parvovirus type 2 (CPV-2) since its discovery, and several lineages and antigenic variants have emerged. Among these, a group of viruses commonly called Asian CPV-2c has recently been reported with increasing frequency in different regions. Currently, its global epidemiology and evolution are essentially unknown. The present work deals with this information gap by evaluating, via sequence, phylodynamic, and phylogeographic analyses, all the complete coding sequences of strains classified as Asian CPV-2c based on a combination of amino acid markers and phylogenetic analysis. After its estimated origin around 2008, this lineage circulated undetected in Asia until approximately 2012, when an expansion in viral population size and geographical distribution occurred, involving Africa, Europe, and North America. Asia was predicted to be the main nucleus of viral dispersal, leading to multiple introduction events in other continents/countries, where infection establishment, persistence, and rapid evolution occurred. Although the dog is the main host, other non-canine species were also involved, demonstrating the host plasticity of this lineage. Finally, although most of the strains showed an amino acid motif considered characteristic of this lineage, several exceptions were observed, potentially due to convergent evolution or reversion phenomena.

## 1. Introduction

In the 1970s, a novel parvovirus capable of infecting canines emerged as a dog pathogen, quickly becoming a major health threat for pets and wild canine populations globally. This virus was named canine parvovirus type 2 (CPV-2) to distinguish it from the only other parvovirus known at that time to infect dogs, the minute virus of canines [1,2]. CPV-2 causes severe enteric disease in affected dogs, which can lead to acute hemorrhagic diarrhea and death, sometimes complicated by myocarditis and lymphopenia-associated immunodepression with consequent super-infections. While the infection can be acquired at any age, the disease is more severe in younger individuals [2,3,4]. CPV-2 infection in cats is similar in its course to what is observed in dogs, but panleukopenia is usually predominant [2,4,5]. Not much is known about the clinical manifestations among wild animals.

CPV-2 belongs to the species *Protoparvovirus carnivoran1* (family *Parvoviridae*, sub-family *Parvovirinae*), together with another virus that circulates among terrestrial felids and mustelids, feline panleukopenia virus (FPV or FPLV) [6,7]. However, before genetic studies clarified that only these two main genetic lineages existed within the species, CPV-2 and FPV were named differently when found in other hosts, such as mink enteritis virus (MEV), raccoon parvovirus (RPV), or blue fox parvovirus (BFPV) [2,8,9,10]. Like all other parvoviruses, viruses within this species are characterized by ~25 nm naked T = 1 icosahedral capsids composed of two viral proteins, VP1 and VP2, which contain a single-stranded DNA molecule of approximately 5 Kb, covalently linked to the main viral non-structural protein, NS1 [6]. The genome includes two gene cassettes—one for the non-structural (NS) and one for the structural (VP) proteins—which, thanks to the alternative splicing of host-transcribed messenger RNAs, produce all open reading frames (ORF) coding for viral proteins. These cassettes are flanked by terminal non-coding regions that fold into hairpin-like structures that are important for viral DNA replication [6].

CPV-2 emerged from an FPV-like virus after acquiring specific mutations that allowed its capsid to bind the transferrin receptor of canine cells, extending its tropism to canines and enabling its fast pandemic spread [1,2,11]. The original CPV-2 strain, which now no longer circulates among dogs, did not possess the ability to infect felines, but the variant that replaced it in the 1980s, known as CPV-2a, (re)gained this ability [1,2]. Since then, CPV-2a and its descendants have been found in several carnivoran hosts, predominantly canids, felids, and mustelids, and even in non-carnivoran mammals [5,8,9,12,13,14,15,16,17]. Despite this difference in host ranges, the genomes of CPV-2 and FPV are approximately 98% identical to each other, and only six amino acid (AA) mutations distinguish their VP2s, while only three amino acid mutations distinguish CPV-2 from CPV-2a. Additionally, mutations at amino acid 300 of VP2 can alter the virus–host tropism, and several different residues have been detected at this position in various members of *Protoparvovirus carnivoran1* infecting different hosts [8,18].

After the emergence of CPV-2a, several new mutations were reported for this virus, seemingly, without shifts in host tropism. The classification of this virus into three antigenic variants depending on the amino acid, featuring position 426 of VP2 (N in CPV-2a, D in CPV-2b, and E in CPV-2c), gained significant consensus [2,19,20]. However, there are contradictory opinions about these mutations’ antigenic and biological relevance, as vaccines remain cross-protective, and the virulence of the three variants seems similar [4,21]. Nonetheless, these and other mutations continuously emerge, and several studies have shown variation in their frequency over the years [2,4,22,23]. Some amino acid mutations, including the one at AA 426, arose in different lineages at various times and places, likely as a result of convergent evolution due to positive selection pressure [24,25]. In fact, the groups of viruses belonging to the same variant are paraphyletic [24]. 

Given these convergences, the presence of one specific phenotypic mutation cannot be used for virus classification. However, the presence of a pattern of mutations, combined with phylogenetic analyses, allows us to more specifically define evolutionary-related strains and monitor their circulation [24,25,26]. This is the case for the so-called “Asian CPV-2c” lineage, a monophyletic group of viruses characterized by a specific set of mutations in both structural and non-structural proteins [25]. This clade was recorded for the first time in Asian countries, where it became more and more prevalent as years passed by, and it was recently introduced to several other countries worldwide, where it is now spreading and locally evolving [12,27,28,29,30,31,32]. However, the understanding of the real success, population dynamics, and spreading patterns of this clade are hindered by sparse and biased diagnostic and sequencing activity. As the success of this clade is intriguing from an evolutionary and epidemiological perspective, in this study, we investigated the origin and evolution of Asian CPV-2c-like viruses, using dedicated statistical and bioinformatic approaches that are less susceptible to sampling structure to gain a deeper understanding of CPV-2 global transmission and evolutionary dynamics.

## 2. Materials and Methods

### 2.1. Datasets

All sequences of members of the species *Protoparvovirus carnivoran1*, available in GenBank as of 2 April 2023, were downloaded and combined with additional sequences deposited later (accession numbers OR463583–OR463704) [32], resulting in a set of 8804 sequences. Three subsets were then created, including full coding (from NS1 start codon plus, at most, 3 AAs to VP2 stop codon minus, at most, 3 AAs), full NS1 (minus, at most, 3 AAs), and full VP2 (minus, at most, 3 AAs) sequences. From the three sets, sequences with ambiguities, frameshift mutations, and premature stop codons were removed. This resulted in three datasets of 733 complete, 901 NS1, and 4765 VP2 coding sequences. For each sequence, the collection host, country, and date were recorded if available, and the sequence name was edited to include this information.

### 2.2. Asian CPV-2c Lineage Definition

Sequence alignments (NS1, VP2, complete genome) were performed with MAFFT [33]. For coding regions, an alignment was initially performed at the amino acid level, and then sequences were back-translated as nucleotides using TranslatorX [34].

Since different methods can lead to slightly different results, especially in the case of high genetic identity and convergent mutations, for each dataset, three phylogenetic trees were reconstructed, and the results were compared. Trees were built using IQ-Tree [35], RAxML [36], and Fasttree2 [37], and using the substitution model with the lowest Bayesian information criterion (BIC) as calculated by JModelTest2 [38]. The Asian CPV-2 lineage was defined based on three expert opinions according to the following:

(1) The presence of the amino acid motif 5A/G, 267Y, 297A, 324I, 370R, 426E, and 440T in VP2 and 630P in NS1. Motif identification was automatically performed using specifically designed R scripts.
(2) Being part of a monophyletic clade, including the majority of strains featured in the above-mentioned markers. This choice was necessary to classify as part of the “Asian CPV-2c” clade some strains that, although originating from an Asian ancestor, lost, because of amino acid toggling and reversion, the peculiar phenotypic pattern.
(3) The strains were identified based on different trees and expert opinions were compared to classify them based on a majority consensus rule: strains fulfilling the second rule in at least two out of three trees built with at least one of the three alignments were classified as “Asian CPV-2c”.

### 2.3. Phylodynamic Analyses

The selected datasets (VP2, NS1, and complete genome) were analyzed to reconstruct several population parameters, including the time to the most recent common ancestor (tMRCA), the evolutionary rate, and viral population dynamics using the Bayesian serial coalescent approach implemented in BEAST 1.10 [39]. The nucleotide substitution model was selected based on a BIC score calculated using JmodelTest [38]. The molecular clock method was selected to calculate the marginal likelihood estimation through path-sampling and stepping-stone methods, as suggested by Baele et al. [40]. A non-parametric Bayesian skygrid [39] was implemented to reconstruct the viral population (relative genetic diversity: effective population size x generation time; Ne x τ) over time. 

Based on the higher sequence number and more representative geographic origin compared with other genomic regions, a discrete-state phylogeographic analysis was also performed as described by Lemey et al., 2009 [41], on the VP2 dataset only. An asymmetric migration model with Bayesian stochastic search variable selection (BSSVS) was implemented, allowing us to identify the most parsimonious description of the spreading process and calculate a BF indicative of the statistical significance of the inferred migration path between areas. Since sampling and sequencing biases are likely, this phenomenon and its impact on analysis results were assessed via subsampling without replacing the available sequences and allowing for a maximum of 8 sequences for each country–date pair. Moreover, this allowed us to create more balanced sequence datasets. 

The history and evolution over time of marker amino acids featuring the Asian CPV-2c lineage were also reconstructed using discrete trait analysis (DTA) in BEAST 1.10. For each analysis, an independent run of 200 million generations was performed. The results were analyzed using Tracer 1.7 [42] after the removal of a burn-in of 20% and accepted only if the estimated sample size (ESS) was greater than 200 and the convergence and mixing were adequate. Parameter estimation was summarized in terms of mean and the 95% highest posterior density (95HPD). Maximum clade credibility (MCC) trees were constructed and annotated using TreeAnnotator (BEAST package). SpreaD3 [43] was used to calculate the BF associated with each migration route. All non-zero transition rates among countries were considered significant when the BF was greater than 10. Additional summary statistics and graphical outputs were generated using homemade R scripts (Team, 2014).

The presence of sites under episodic diversifying selection was assessed using MEME [44], implemented in HyPhy [45], while the positive pervasive selection was evaluated using FEL [46] and FUBAR [47], implemented in the same program. The statistical significance was set at *p* < 0.05 and the posterior probability at >0.9.

## 3. Results

### 3.1. Details of the Sequences Identified as Belonging to the Asian CPV-2c Lineage

**Figure 1:**
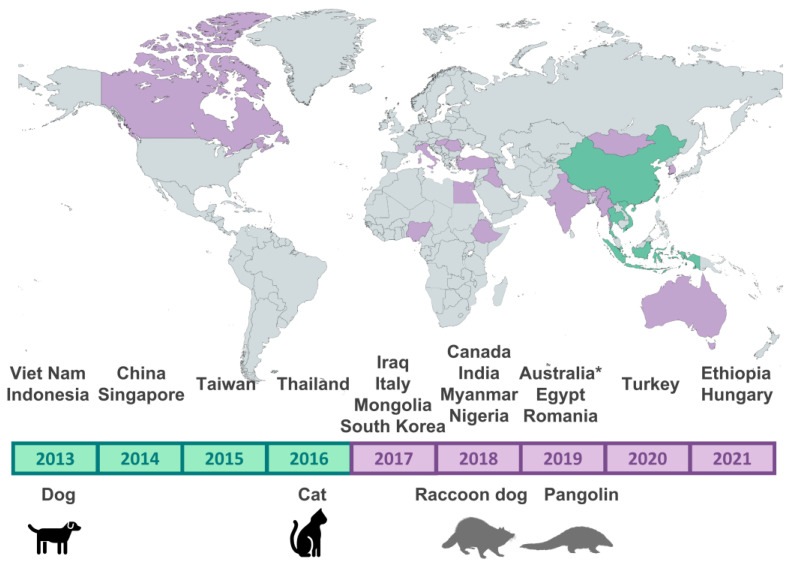
Temporal, host, and country distribution of sequences belonging to the Asian CPV-2c lineage annotated in GenBank. In the map at the top, the countries where the earliest sequences (2013–2016) were identified are indicated in green, while those where the viruses were found later (2017–2021) are in purple. The gray on the map indicates no sequences reported as of yet. In the lower part, countries (top) and hosts (bottom: in black, domestic animals, and in gray, other animals) are indicated in correspondence to the years when they were detected for the first time. The map was created with Mapchart.net ^©^. * Classified based on NS1.

After searching the NCBI sequence database, a total of 917 sequences belonging to the Asian CPV-2c lineage were identified. The earliest sequences (2013) originated from Asian (Vietnamese and Indonesian) dogs, but since 2017, viruses belonging to this clade started to be detected on other continents as well (Figure 1), having been recorded in dogs but also cats (*Felis silvestris catus*), raccoon dogs (*Nyctereutes procyonoides*) (Republic of Korea), and pangolins (*Manis pentadactyla*) (Taiwan and China). In total, 872 of these strains could be classified as CPV-2c, 7 as CPV-2b, and 3 as CPV-2a, depending on the amino acid present at residue 426 of VP2. Supplementary Table S1 details how many sequences were identified for each year, country, host, and antigenic type, while the Supplementary File provides the accession numbers and a description of each identified sequence.

**Figure 2:**
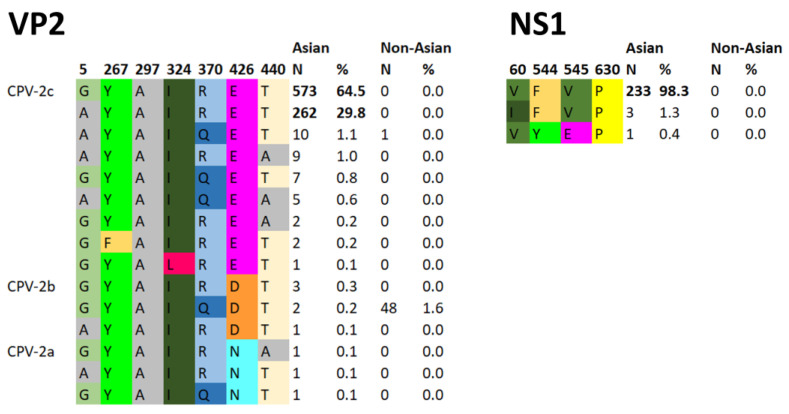
Key amino acids at specific residues in VP2 (**left**) and NS1 (**right**), defining Asian CPV-2c strains. In each panel, the amino acid position is indicated at the top, while the number (N) of times and percentages (%) that these sequences were found in the CPV-2c Asian (Asian) clade and in all other CPV-2 strains (non-Asian) are indicated on the right.

By analyzing the amino acid residues that were used in the literature to determine whether a strain belonged to this clade (60V, 544F, 545V, and 630P in NS1 and 5G, 267Y, 297A, 324I, 370R, 426E, and 440T in VP2) [25], we observed that, while none of these mutations are unique to this clade, and not all sequences in this clade possess all of these mutations, specific combinations of mutations were significantly associated with this clade and not other clades (Figure 2). Specifically, NS1-630P was found in all sequences belonging to the Asian CPV-2c lineage, and VP2 324I, 297A, 267Y, 370R, 426E, and 440T were simultaneously present in 94.3% of sequences in this clade. Interestingly, the monophyletic Asian lineage also included a few CPV-2b (426D) and CPV-2a (426N) strains (see Section 3.4). A summary of amino acid residues present in all other strains is available in Supplementary Figure S1.

### 3.2. Phylodynamic Analyses

**Figure 3:**
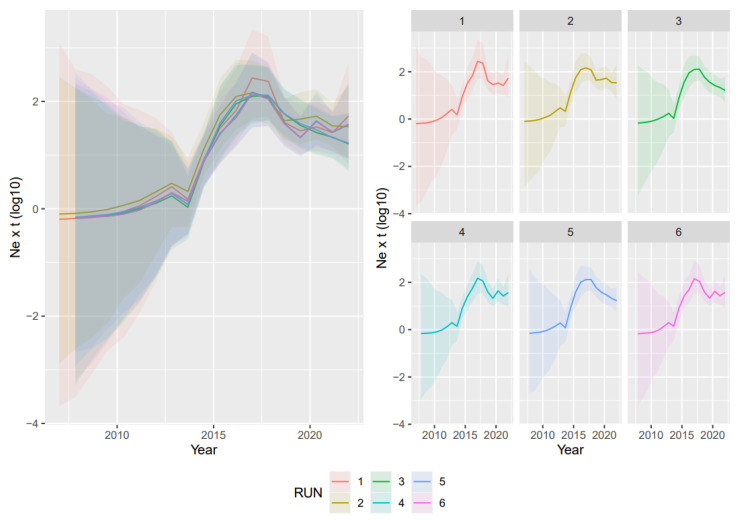
Relative genetic diversity (Ne x t) of the Asian CPV-2c lineage over the years. The results of the six independent runs have been color-coded. Mean, median, and upper and lower 95HPD values are reported for each run on the right and superimposed on the left.

The VP2-based analyses led to concordant results regardless of the randomly generated dataset. The average evolutionary rate was 4.62 × 10^−4^ subs/site/year (95HPD: 2.21 × 10^−4^–7.78 × 10^−4^), and the tMRCA was estimated in 2006.43 (95HPD: 1972.41–2009). The reconstruction of the population dynamics allowed us to identify three main phases: the first one, right after the origin of the lineage, featured a substantially stable population size; this was followed by a rapid increase in diversity approximately in the period 2012–2017 and, finally, after the population peak, by a slow decline (Figure 3).

Comparable results were obtained for the NS1 dataset, whose evolutionary rate was estimated to be 4.87 × 10^−4^ subs/site/year (95HPD: 2.26 × 10^−4^–6.94 × 10^−4^), and the tMRCA was 2008.97 (95HPD: 1969.26–2013). Although it had a relatively broad uncertainness, a stabler population size was inferred using NS1, with minor fluctuations occurring over the last decade (Supplementary Figure S2).

Finally, the estimated evolutionary rate of the complete genome was 6.64 × 10^−4^ subs/site/year (95HPD: 4.22 × 10^−4^–1.24 × 10^−3^), and the tMRCA was 2010.19 (95HPD: 2006.4–2012.56) (Supplementary Figure S3). A relatively constant population size, with minor fluctuations, was also reconstructed. 

### 3.3. Phylogeographic Analyses

**Figure 4:**
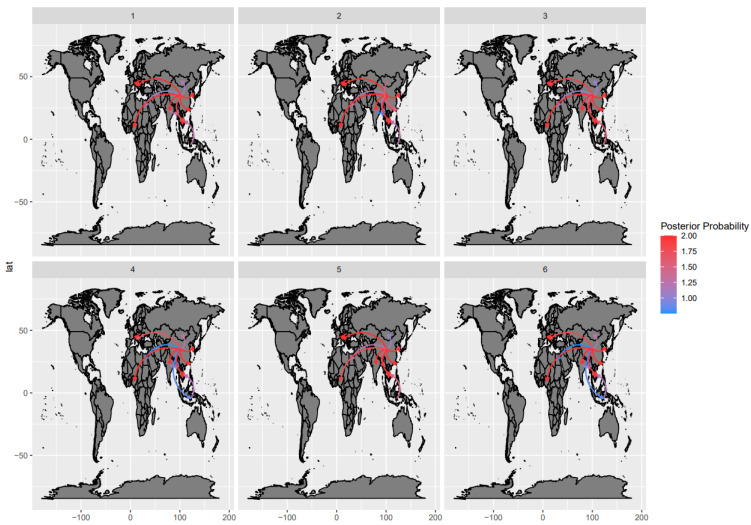
Well-supported migration paths (i.e., BF > 10) of strains belonging to the Asian CPV-2c lineage among countries are depicted as edges whose colors are proportional to the base-10 logarithm of the migration rate. The location of each country is matched with its centroid. The results of the 6 independent analyses are reported in different panels.

The phylogeographic analyses performed on randomly and independently generated datasets, despite minor variations due to the differences in strains included, highlighted an essentially common pattern (Figure 4). Almost all statistically supported migration rates pointed to centrifugal spreading and multiple, independent introductions from Asia (China) to other Asian, African, and European countries (Figure 4).

**Figure 5:**
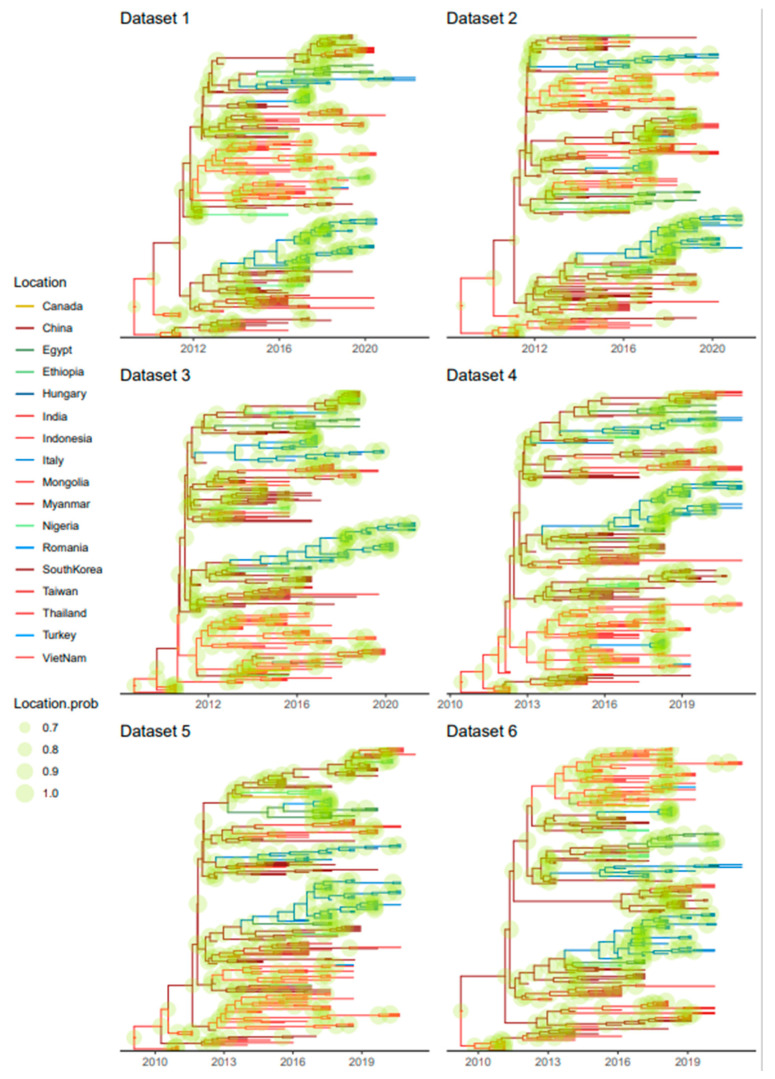
Maximum clade credibility trees based on the Asian CPV-2c lineage VP2 dataset. The results of the phylogeographic analyses are reported with different colors. Tips and branches are color-coded according to the collection country or the one estimated with the higher posterior probability, respectively. Countries are reported in different shades of color featuring the same continent. Node size is proportional to the posterior probability of the inferred locations. The results of different datasets are reported in different panels. A comparable figure with reported tip names is provided in Supplementary Figure S4.

Specifically, after an Asian origin, potentially in Indonesia or China, the “Asian CPV-2c” lineage persisted in the region until approximately 2012, when new continents started to be involved, including European and, essentially in the same period, African countries. Multiple independent introductions occurred from Asian countries to both Europe and Africa. These events were followed by local persistence and evolution (Figure 5). In addition to within-continent clustering, the tendency of strains collected in the same country to cluster together was apparent. Nevertheless, the clustering of strains sampled in different countries of the same continent and the circulation of highly divergent strains in the same country were observed (Figure 5 and Figure S4).

### 3.4. Marker Amino Acid Evolution

**Figure 6:**
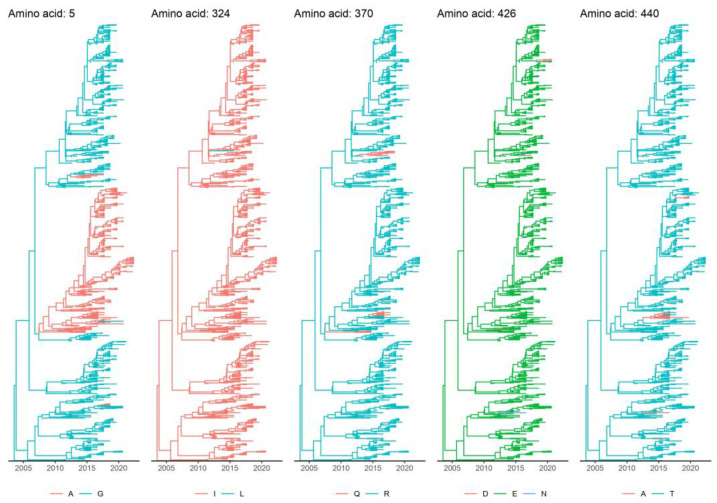
Maximum clade credibility trees based on the Asian CPV-2c lineage VP2 dataset. The results of the DTA analysis—reconstructing the evolution, over time, of the amino acids traditionally used as markers to define Asian CPV-2c strains—are reported in different panels. Tips and branches are color-coded according to the detected amino acid or the one estimated with the higher posterior probability, respectively.

Although the Asian CPV-2c lineage has been traditionally defined based on the presence of amino acid markers in specific VP2 positions, a certain variability was observed among its strains. To understand whether this feature was ascribable to basal branches (i.e., ancestral strains not fully displaying the final phenotype) or to reversion/toggling phenomena, the evolution of variable marker positions was reconstructed through a DTA. In all instances, the variability affected recent, terminal branches, providing evidence for the second hypothesis. Although single-terminal branches were mainly affected, some clades with 370Q persisted for a certain period. A minor recent clade with European (Italy and Hungary) sequences showing 426D was also present (Figure 6).

MEME analysis detected amino acids 5, 119, 354, 426, and 542 (including two Asian CPV-2c markers) under episodic diversifying selection at the set significance level (*p* < 0.05). FEL and FUBAR detected amino acids 5 and 440 (both Asian CPV-2c markers) and 5, 13, 440, and 426 (three of which are Asian CPV-2c markers) under diversifying selection, respectively. In NS1, AAs 492 and 665 were detected under diversifying selection with FUBAR, while AAs 647, 664, and 665 were detected with MEME.

## 4. Discussion

Since it emerged as a devastating dog pathogen, CPV-2 has demonstrated a high evolutionary rate, which has led to the emergence of remarkable genetic and phenotypic variability [10]. For a long time, the primary research interest was the description and characterization of phenotypic/antigenic variants, which are defined based on the presence of peculiar combinations of amino acidic markers in specific positions of the VP2 protein [1,2]. CPV-2a, -2b, and -2c are the most widely recognized variants, whose definition is largely shared by most research groups [2,19,20]. Over time, different studies have reported a plethora of other potential genetic variants, which have shown inconsistent epidemiological success. Most recently, an increase in the detection frequency of the so-called “Asian CPV-2c” lineage was observed [27,28,29,30,32,48]. However, no systematic study has been performed to evaluate its molecular epidemiology and evolution on a broader scale. The present work deals with this knowledge gap.

According to our models, the origin of the Asian CPV-2c lineage was estimated to be approximately in 2008 in Asian countries. Thereafter, it likely circulated in the area, undetected or unreported for years, progressively but slowly increasing in population size in accordance with what was previously reported [49]. Comparably to what was previously reported for other CPV-2 variants, the Asian lineage featured an overall high evolutionary rate [10,48,49,50]. A major change in this pattern was observed after 2012 when an abrupt expansion occurred. This period coincided with the first official description of Asian CPV-2c strains, whose detection was facilitated by an increase in infection (and likely disease) prevalence. The first available sequences were, in fact, from samples collected in 2013 (dogs from Vietnam and Indonesia), 2014 (dogs from China and Singapore), and 2015 (dogs from Taiwan and China). However, from 2016 onward, an increase in the number of the sequences submitted yearly to GenBank was observed, likely corresponding to more sustained viral circulation. This finding confirms the huge spreading potential that has characterized the history of CPV-2 and, thus, the need for more effective and systematic monitoring activities to promptly detect and effectively react to emerging viruses. In fact, the increase in population size also mirrored the viral introduction to new areas of the world, and strains belonging to this lineage have been reported particularly in Asia [30,51,52], Europe [25,27,29,53], and Africa [31,54,55], although there is also a report from North America [12]. Furthermore, in recent years, an increasing number of studies from Asia have highlighted not only the progressive spread of the Asian CPV-2c lineage but also the progressive replacement of the circulating CPV-2 strains by this lineage, as if it had potentially greater viral fitness [56,57,58,59]. Similarly, a strong expansion of this lineage, which replaced other locally circulating CPV-2c variants soon after its first occurrence, was also recently observed in Italy [32]. Although a biological advantage of the CPV-2c Asian lineage over other CPV-2 variants could be speculated, no definitive proof is present, opening interesting new research fields regarding this topic.

The higher infection rates in Asian countries, likely also favored by lower vaccination coverage, could have increased the likelihood of strain exportations. Epidemiological links mediated by both human and animal movement between Asia and Europe have been proven for several companion animal infections, including CPV-2 [28,60,61,62,63]. Similarly, the rising political, economic, and cultural interactions occurring between Asian (especially China) and African countries might have favored the introduction of this lineage, as previously suggested [48,55].

Alternatively, the increase in the viral population size could have followed the introduction into new areas, allowing this lineage to benefit from a broader host population. Vaccination is especially rare in African dog populations [48], which may have favored viral expansion. Although vaccination rates are much higher in Europe, understanding whether immunity induced by other strains confers less protection against this new lineage is intriguing and requires further investigation. The final stabilization and slow decline of the viral population size modeled in the present study could support this hypothesis, reflecting the progressive establishment of lineage-specific immunity. A combination of the two phenomena should also not be excluded. 

The spreading dispersal pattern analysis suggests Asia is the central nucleus of viral spread, mediating multiple independent introductions to other continents, followed by successful infection establishment, persistence, and local evolution. Although a tendency toward country-based clustering was present, evidence of within-continent dispersal was also observed, although with higher among-dataset variability. Accordingly, none of those connections were statistically significant. Overall, local persistence and spreading were the dominant phenomena, as we also observed elsewhere [32], while transboundary dispersal was more sporadic. This pattern, featuring multiple independent introduction events as a consequence of inter- and intracontinental migrations, followed by local differentiation and competition between populations, is a hallmark of CPV-2 epidemiology [64,65,66] and reveals the remarkable evolutionary consequences of poorly constrained viral circulation.

Besides dogs, infections with this lineage have also been reported multiple times in cats (Thailand and China), although FPV is still the dominant virus in felids [67,68,69]. Additionally, Asian CPV-2c strains have also been observed in wild canids, such as in one raccoon dog (Republic of Korea), and even in non-carnivoran hosts, including two pangolins (China and Taiwan) that likely died because of the infection [15,17]. This indicates that these viruses are capable of cross-species transmission, even to non-carnivorans, and should be monitored because they pose a potential danger to wildlife. Whether this lineage emerged in wild animals and then spilled over to dogs, as happened with CPV-2a strains [70], or originated in dogs and then spilled over to other animals remains to be established. 

The above-mentioned pattern could be at least partially explained by the biased and unbalanced number of sequences (especially complete genome sequences) among countries, which might have led to an overestimation of the contribution of more represented countries. For example, while several investigations of the molecular epidemiology of CPV-2 have been performed in Europe and Asia, almost no studies have investigated viral diversity in North America, and very few studies have assessed viral presence among wild animals. Nonetheless, the substantial agreement between the randomly generated datasets seems to contradict this proposition and supports the robustness of the obtained findings. However, the absence of a proper sampling design necessitates caution in results interpretation. While the overall patterns can be considered reliable, specific county pair connections must not be overstated. The long branch length separating several clades likely conceals additional spatial movements between multiple locations, and intermediate links between country pairs might have been lost because of limited diagnostic and sequencing activity.

While marker positions are still often used to classify CPV-2 strains, the incongruence between genetic- and phenotypic-based clustering has also been demonstrated by other authors [24,66]. Strains with a separate evolutionary history can share the same amino acid because of convergent evolution, while reversion phenomena can cause closely related strains to show different phenotypic features. Such phenomena feature in the Asian CPV-2c lineage too. Although not common since >94% of the sequences from this lineage present the same amino acids at key residues (namely, 630P in NS1 and 324I, 297A, 267Y, 370R, 426E, and 440T in VP2), some of the strains classified in this lineage revealed a different phenotypic pattern. In all instances, the amino acid mutations affected terminal branches rather than basal ones, demonstrating episodes of reversion. Establishing whether such mutations can provide an evolutive advantage or are an incidental finding is challenging. Since mostly single branches were involved, low-fitness, epidemiological dead ends are likely. However, some long-lasting clades harboring 5A, 370Q, and 440A were observed. Of note, a phenotypically CPV-2b (AA 426D) clade was observed, including Italian strains from 2022 and a Hungarian one sampled in 2021 [25,32,71]. A similar mutation also independently affected three strains from China sampled since 2016. Two strains sampled in 2017 and 2018 from China and one from India independently acquired 426N. Since positions 5, 426, and 440 were detected under diversifying selection, a potential beneficial effect and fitness advantage induced by phenotypic variability might be speculated. Whether this is ascribable to immune pressures or other forces remains to be established. Biological meaning aside, our results again stress the unreliability of marker-based classification and the importance of integrating sequence and phylogenetic analyses when studying the molecular epidemiology of this virus, as we highlighted in previous studies [25,26,66,72].

## 5. Conclusions

The present study reconstructs the history, evolution, and spread of the Asian CPV-2c lineage from its origin to the present day. After likely relatively long and undetected circulation in Asia, it was able to spread worldwide, increasing its prevalence and relative genetic diversity. Asia was estimated to be the central nucleus of viral dispersal, mediating multiple independent introductions to other continents, followed by successful infection establishment, local persistence, and evolution. A certain phenotypic variability was observed within this clade, resulting from a combination of high mutation rates and selective pressure action. Our results stress one more time the need for systematic epidemiological studies, monitoring, and reporting for CPV-2 activity and diversity to promptly detect new CPV-2 variants and lineages, whose behavior is currently hardly predictable, creating the best conditions for their control.

## Data Availability

The sequences used in this study are all available in GenBank under the accession numbers listed in the Supplementary File.

## Supplementary Materials

The following supporting information can be downloaded at https://www.mdpi.com/article/10.3390/v15091962/s1: Table S1: Number of sequences within the Asian CPV-2c lineage identified in each country, year, and host and for each variant. Figure S1: Key amino acids at specific residues in VP2 and NS1, defining Asian CPV-2c viruses in strains that do not belong to the Asian CPV-2 lineage. Figure S2: Relative genetic diversity (Ne x t) of the Asian CPV-2c lineage over the years, estimated using the NS1 gene. Figure S3: Relative genetic diversity (Ne x t) of the Asian CPV-2c lineage over the years, estimated using the complete genome. Figure S4: Maximum clade credibility trees based on the Asian CPV-2c lineage VP2 dataset.
