# Supplementary material for: Not Asian Anymore: Reconstruction of the History, Evolution, and Dispersal of the “Asian” Lineage of CPV-2c
Source: Viruses. 2023 Sep 20;15(9):1962. doi: 10.3390/v15091962 (PMC10535194; doi:10.3390/v15091962)
Supplement: Supplementary file 1 [file viruses-15-01962-s001.zip › Supplementary Figure S4.pdf]

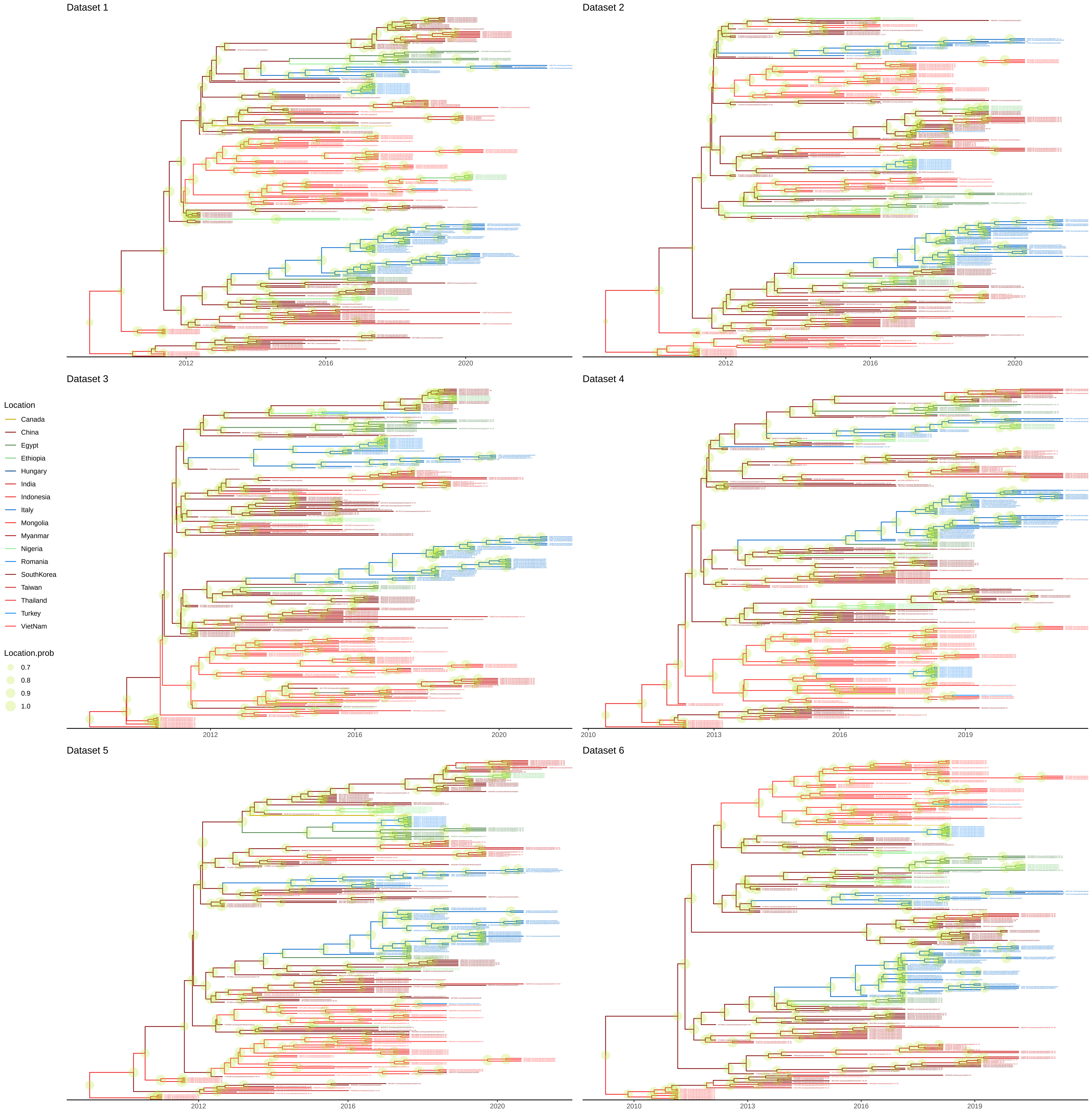
Dataset 1
2012
2016
2020
Dataset 2
2012
2016
2020
Dataset 3
2012
2016
2020
Dataset 4
2010
2013
2016
2019
Dataset 5
2012
2016
2020
Dataset 6
2010
2013
2016
2019
Location
Canada
China
Egypt
Ethiopia
Hungary
India
Indonesia
Italy
Mongolia
Myanmar
Nigeria
Romania
SouthKorea
Taiwan
Thailand
Turkey
VietNam
Location.prob
0.7
0.8
0.9
1.0
