# Supplementary material for: Not Asian Anymore: Reconstruction of the History, Evolution, and Dispersal of the “Asian” Lineage of CPV-2c
Source: Viruses. 2023 Sep 20;15(9):1962. doi: 10.3390/v15091962 (PMC10535194; doi:10.3390/v15091962)
Supplement: Supplementary file 1 [file viruses-15-01962-s001.zip › SupplementaryMaterial.pdf]

Not Asian Anymore: Reconstruction of the History, Evolution, and Dispersal of the "Asian" lineage of CPV-2c

Giovanni Franzo, Francesco Mira, Giorgia Schiro and Marta Canuti

Table S1. Number of sequences within the Asian CPV-2c lineage identified in each country, year, host, and for each variant

Country	N sequences	Country	N sequences	Country	N sequences
Australia	1	Indonesia	7	Singapore	3
Canada	1	Iraq	2	South Korea	10
China	569	Italy	137	Taiwan	6
Egypt	14	Mongolia	1	Thailand	15
Ethiopia	4	Myanmar	15	Turkey	1
Hungary	1	Nigeria	18	Viet Nam	70
India	25	Romania	10	Unknown	7
**Year**	**N sequences**	**Year**	**N sequences**	**Year**	**N sequences**
2013	8	2018	119	2018-2019	16
2014	28	2019	256	2020-2021	1
2015	6	2020	201	Unknown	22
2016	48	2021	83		
2017	102	2022	44		
**Host**	**N sequences**	**Variant**	**N sequences**		
Cat	12	CPV-2a	4		
Dog*	895	CPV-2b	7		
Pangolin	3	CPV-2c	872		
Raccoon dog	1	Unknown	35		
Unknown	6				

* labelled as dog, canine, canis, or puppy.

VP2

CPV-2c

5	267	297	324	370	426	440	N	%
A	F	A	Y	Q	E	T	**450**	**88.2**
A	F	A	Y	Q	E	A	50	9.8
A	F	N	Y	Q	E	T	2	0.4
A	F	A	Y	Q	E	S	2	0.4
F	F	A	Y	Q	E	T	1	0.2
A	F	A	Y	R	E	T	1	0.2
A	Y	A	Y	Q	E	T	1	0.2
A	Y	A	Y	Q	E	A	1	0.2
G	F	A	Y	R	E	T	1	0.2

CPV-2a

5	267	297	324	370	426	440	N	%
A	Y	A	I	Q	N	A	**657**	**38.5**
A	F	A	Y	Q	N	T	**398**	**23.3**
A	F	A	I	Q	N	T	**249**	**14.6**
A	F	S	Y	Q	N	T	**191**	**11.2**
A	F	A	Y	Q	N	A	51	3.0
A	F	A	L	Q	N	T	33	1.9
A	F	A	Y	Q	N	S	28	1.6
A	Y	A	I	Q	N	T	22	1.3
G	Y	A	Y	Q	N	T	11	0.6
A	F	A	I	Q	N	A	8	0.5
G	Y	A	I	Q	N	A	7	0.4
G	F	A	I	Q	N	T	7	0.4
A	F	N	Y	Q	N	T	6	0.4
A	F	A	I	R	N	T	6	0.4
A	F	N	Y	Q	N	A	4	0.2
A	F	A	L	Q	N	A	4	0.2
G	F	A	Y	Q	N	T	4	0.2
A	F	A	F	Q	N	T	4	0.2
A	Y	A	Y	Q	N	T	3	0.2
A	F	A	N	Q	N	T	3	0.2
A	F	A	F	Q	N	A	1	0.1
A	F	S	I	Q	N	T	1	0.1
A	Y	A	T	Q	N	A	1	0.1
T	F	S	Y	Q	N	T	1	0.1
G	F	S	Y	Q	N	T	1	0.1
P	Y	A	I	Q	N	A	1	0.1
A	F	S	I	Q	N	A	1	0.1
A	F	N	I	Q	N	T	1	0.1
A	Y	A	I	K	N	A	1	0.1
G	Y	S	Y	Q	N	T	1	0.1
V	F	A	I	Q	N	T	1	0.1

CPV-2b

5	267	297	324	370	426	440	N	%
A	F	A	Y	Q	D	T	**434**	**54.3**
A	Y	A	I	Q	D	A	**162**	**20.3**
G	Y	A	Y	Q	D	T	43	5.4
A	F	N	L	Q	D	T	26	3.3
A	Y	A	Y	Q	D	T	24	3.0
A	F	S	Y	Q	D	T	22	2.8
A	F	N	Y	Q	D	T	13	1.6
A	F	A	Y	Q	D	S	6	0.8
A	F	A	L	Q	D	T	5	0.6
A	F	A	I	Q	D	A	3	0.4
A	F	A	I	Q	D	T	2	0.3
G	Y	A	I	Q	D	A	2	0.3
A	F	A	F	Q	D	A	2	0.3
A	F	A	Y	Q	D	A	2	0.3
A	F	N	I	Q	D	T	2	0.3
A	Y	T	I	Q	D	T	1	0.1
A	L	A	Y	Q	D	T	1	0.1
A	Y	A	N	Q	D	T	1	0.1
A	Y	A	Y	Q	D	A	1	0.1

NS1

60	544	545	630	N	%		
I	Y	E	L	**423**	**63.4**	**423**	**63.4**
I	F	E	L	**114**	**17.1**	**114**	**17.1**
I	F	V	L	66	9.9	66	9.9
I	Y	Q	L	38	5.7	38	5.7
V	Y	E	L	9	1.3	9	1.3
I	F	E	P	8	1.2	8	1.2
I	Y	V	L	5	0.7	5	0.7
I	Y	K	L	2	0.3	2	0.3
V	F	E	L	1	0.1	1	0.1
I	F	Q	L	1	0.1	1	0.1

Figure S1. Key amino acids at specific residues in VP2 (left and top-right) and NS1 (bottom-right) defining Asian CPV-2c viruses in strains that do not belong to the Asian CPV-2 lineage. In each panel, the amino acid position is indicated on top, while the number (N) of times and percentages (%) these sequences were fund each group are indicated on the right

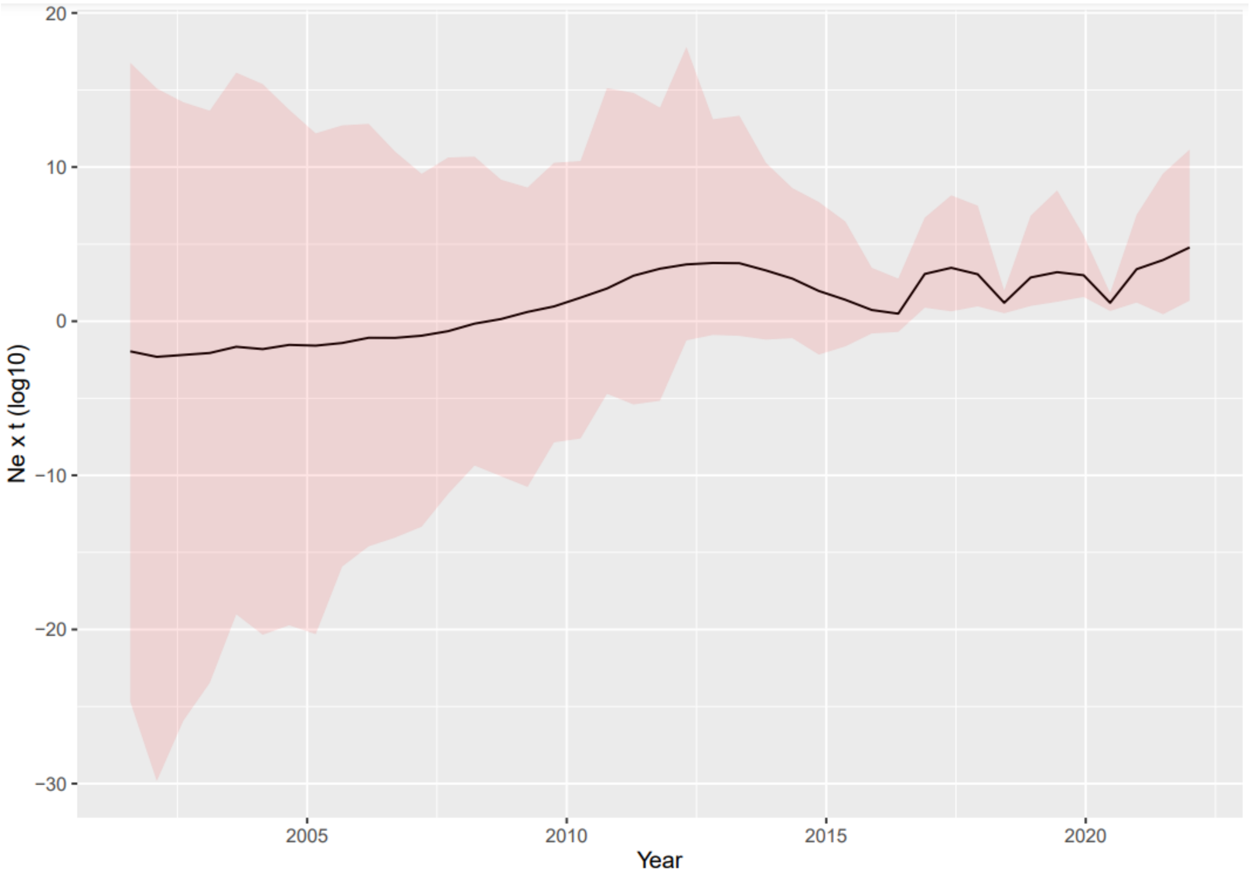

Figure S2. Relative genetic diversity (Ne x t) of the Asian CPV-2c lineage over the years estimated using the NS1 gene. Mean, median, and upper and lower 95HPD values are reported

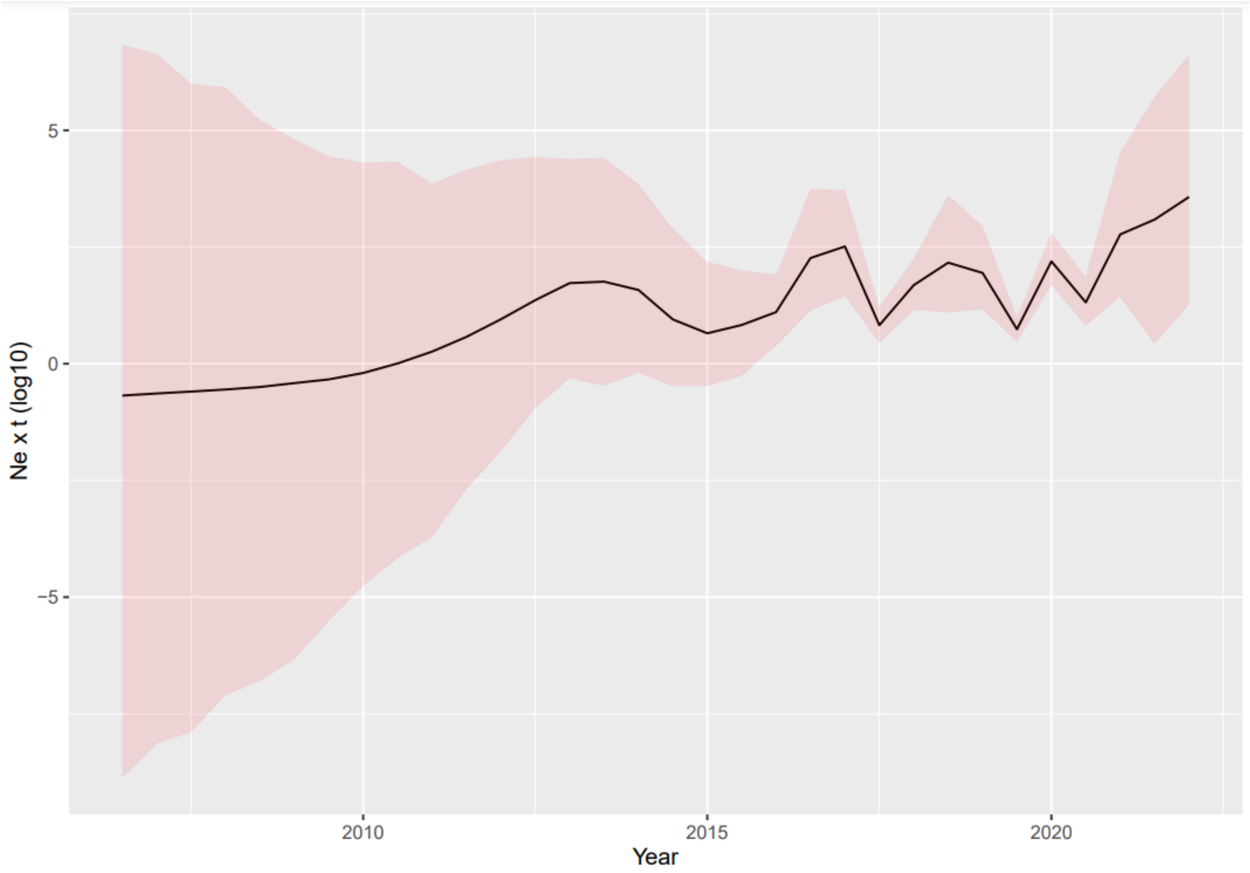

Figure S3. Relative genetic diversity (Ne x t) of the Asian CPV-2c lineage over the years estimated using the Complete genome. Mean, median, and upper and lower 95HPD values are reported

Figure S4. Maximum clade credibility trees based on the Asian CPV-2c lineage VP2 dataset. The results of the phylogeographic analyses are reported with different colors. Tips and branches have been color-coded according to the collection country or the one estimated with the higher posterior probability, respectively. Countries are reported in different nuances of the color featuring the continent. Node size is proportional to the posterior probability of the inferred locations. The results of different datasets are reported in different panels. [Figure in a separate file]
